# Targeting Oxidative Stress Reduction and Inhibition of HDAC1, MECP2, and NF-kB Pathways in Rats With Experimentally Induced Hyperglycemia by Administration of *Thymus marshallianus* Willd. Extracts

**DOI:** 10.3389/fphar.2020.581470

**Published:** 2020-09-23

**Authors:** Alexandra C. Sevastre-Berghian, Irina Ielciu, Andrei Otto Mitre, Gabriela A. Filip, Ilioara Oniga, Laurian Vlase, Daniela Benedec, Ana-Maria Gheldiu, Vlad A. Toma, Bianca Mihart, Andra Mihuţ, Ioana Bâldea, Diana Olteanu, Irina C. Chis, Simona V. Clichici, Daniela Hanganu

**Affiliations:** ^1^Department of Physiology, Faculty of Medicine, “Iuliu Haţieganu” University of Medicine and Pharmacy, Cluj-Napoca, Romania; ^2^Department of Pharmaceutical Botany, Faculty of Pharmacy, “Iuliu Haţieganu” University of Medicine and Pharmacy, Cluj-Napoca, Romania; ^3^Department of Pharmacognosy, Faculty of Pharmacy, “Iuliu Haţieganu” University of Medicine and Pharmacy, Cluj-Napoca, Romania; ^4^Department of Pharmaceutical Technology and Biopharmacy, “Iuliu Haţieganu” University of Medicine and Pharmacy, Cluj-Napoca, Romania; ^5^Department of Molecular Biology and Biotechnology, Faculty of Biology and Geology, Babeş-Bolyai University, Cluj-Napoca, Romania; ^6^Department of Biochemistry and Experimental Biology, Institute of Biological Research, Cluj-Napoca, Romania; ^7^Department of Molecular and Biomolecular Physics, NIRD for Isotopic and Molecular Technologies, Cluj-Napoca, Romania

**Keywords:** *Thymus marschallianus*, polyphenols, HDAC1, MECP2, NF-kB, oxidative stress, experimentally induced hyperglycemia, antioxidant

## Abstract

The effects of two lyophilized extracts obtained from the aerial parts of *Thymus marschallianus* Willd. and harvested from wild flora (TMW) and obtained from culture (TMC) were evaluated in Wistar rats with experimentally induced hyperglycemia. The hyperglycemia was induced by streptozotocin (STZ) administration and the obtained results were evaluated in comparison for TMW and TMC. The polyphenolic composition of extracts was evaluated by spectrophotometrical and LC-MS methods. *In vitro* antioxidant capacity assays (DPPH, FRAP, EPR) were performed in order to preliminary establish the ability of tested samples to protect against free radical induced damage. Afterwards, the effects of these extracts were assessed *in vivo* on rats with experimental-induced hyperglycemia. Oxidative stress biomarkers (e.g. malondialdehyde—MDA), phosphorylated transcription factor subunit of nuclear kappaB (NF-kB) p65, methyl CpG binding protein (MECP) 2 and histone deacetylase 1 (HDAC1) expressions in hippocampus and frontal lobe were assessed. Open Field Test (OFT) and Elevated Plus Maze (EPM) were conducted on tested animals. Malondialdehyde (MDA) levels and HDAC1and MeCP2 expressions increased significantly in hippocampus (p<0.05) and frontal lobe (p<0.001) of diabetes group compared to the control group in parallel with decreasing of GSH/GSSG ratio. TMW and TMC administration reduced blood glucose levels and diminished lipid peroxidation, HDAC1 expression and enhanced antioxidant capacity in frontal lobe. TMW improved central locomotion of rats, increased phospho-NFkB p65 and diminished MECP2 expressions in hippocampus. Both tested samples exerted a beneficial effect by increasing the antioxidant defense. Our findings indicate that the administration of these extracts might represent a good option in the treatment of diabetes and its complications.

## Introduction

Diabetes mellitus (DM) is a chronic disease, characterized by hyperglycemia, due either to impairment of insulin sensitivity, insulin secretion or both. It is a major problem for public health and is responsible for a variety of complications such as an increased risk of cardiovascular disorders, nephropathy, retinopathy, neuropathy and cognitive dysfunction (World Health Organization, 2016). According to the International Diabetes Federation, in 2019, a total of 463 million people was estimated to be living with diabetes, namely 9.3% of the global adult population (20–79 years), a number that is expected to increase in the future (International Diabetes Federation, 2019; Saeedi et al., 2019). Elderly adults (≥ 60–65 years old) are at a higher risk of macrovascular complications and common geriatric syndromes, such as visual and cognitive impairment, depression, urinary incontinence and pain, as compared to younger people with diabetes (Chentli et al., 2015). Cognitive functions, such as attention, memory, learning, motor speed, visuoconstruction, somatosensory examination, motor strength, and executive functions are negatively influenced by poor glycemic control. Even though the exact link between diabetes mellitus and cognitive impairment is not fully elucidated, some authors have revealed that hyperglycemia, vascular disease (Fusco et al., 2019; Impellizzeri et al., 2019), hypoglycemia, insulin resistance, and amyloid deposition can be associated with cognitive dysfunction, especially in elderly people (Kodl and Seaquist, 2008). The efficiency of insulin and antihyperglycemic agents (e.g. biguanides, glinides and sulfonylureas) is well known. However, their administration may lead to numerous side effects (impairment of gastrointestinal function, hypoglycemia, or liver dysfunction) (Nozaki et al., 2017). Considering the possible side effects of classical medication and the hypothesis that hyperglycemia is associated with the development of diabetic complications, the need of an alternative therapy that may exert hypoglycemic activity, is a good strategy for the management of this disease (Kodl and Seaquist, 2008; Nozaki et al., 2017).

Scientific evidence maintains that hyperglycemia can lead to reactive oxygen species (ROS) production, in diabetic patients, through mitochondrial respiratory chain enzymes, xanthine oxidases, lipoxygenases, cyclooxygenases, nitric oxide synthases, and peroxidases (Balaban et al., 2005; Volpe et al., 2018). Moreover, clinical and experimental studies indicate that high sugar levels alter some signaling pathways, such as diacylglycerol, the activation of protein kinase C (PKC) and NADPH-oxidase system, thus producing oxidative stress, the formation of advanced glycation end products (AGEs), the secretion of the pro-inflammatory cytokines and cellular death (Nogueira-Machado and Chaves, 2008; Volpe et al., 2018). Additionally, it is proved that the NFkB, known for its role in inflammation, may be activated by hyperglycemia. There is scientific evidence that sustain the involvement of NFkB in synaptic plasticity, learning and memory, also mentioning that spontaneous synaptic transmission, short- and long-term synaptic plasticity, learning and memory-related behaviors are regulated by the MeCP and HDAC activity (Patel and Santani, 2009; Kavalali et al., 2011; Snow and Albensi, 2016). Moreover, the free radicals including superoxide anion from mitochondria can amplify the metabolic damage by the activation of sorbitol-aldose reductase pathways (polyol pathway) and hexosamine pathways and by the inactivation of two protective enzymes: endothelial nitric oxide synthase (NOS) and prostacyclin synthase. This further promotes inflammation resulting in a vicious cycle where hyperglycemia stimulates inflammation. ROS can produce the damage of cellular organelles and enzymes, increased lipid peroxidation, and development of insulin resistance (Balaban et al., 2005; Chentli et al., 2015; Volpe et al., 2018).

Due to their ability of scavenging ROS, the antioxidants can be used as adjuvant therapy in DM, to diminish the damages induced by oxidative stress. Among the most well-known antioxidants are medicinal plants, which exhibit their antioxidant capacity due to their chemical composition, especially to their abundance in polyphenols, which have proven along time efficient antioxidant properties (Nozaki et al., 2017).

*Thymus* (thyme) is one of the most important genus belonging to the Lamiaceae family (Niculae et al., 2019). This family is known worldwide for its traditional uses, in the treatment of different pathologies, being also used since ancient times for their importance as aromatic plants (Nabavi et al., 2015), that are assigned to their composition in essential oils (Tohidi et al., 2019) and phenolic compounds (Raudone et al., 2017). *Thymus marschallianus* Willd. (TM) is a species belonging to the *Thymus* genus which presents numerous chemovarieties: thymol, carvacrol, thymol/carvacrol, geraniol and carveole, that are related to the composition of its essential oils (Salehi et al., 2019). Besides the important number of volatile compounds, the species is also known for its composition in phenolic compounds, which are also responsible for its biological activity (Raudone et al., 2017). Among these compounds, the most important appears to be rosmarinic acid (RA), which is found in large amounts in the flowering aerial parts of the species (Niculae et al., 2019). This phenolic acid is responsible for numerous protective biological activities (Nunes et al., 2017), including the antidiabetic potential and antioxidant capacity (Nunes et al., 2017; Ngo et al., 2018; Nadeem et al., 2019). The two biological effect are even more important as they can can be highly connected in mechanisms. In this context, the evaluation of the protective activity against the hyperglycemic stress of the *T. marschallianus* Willd. species appears to be important, especially as it can be further exploited in the preparation of phytomedicines having important activity in the treatment of diabetes mellitus and its related complications.

Taken all these into consideration, the objective of this study was to evaluate the effect of two *T. marschallianus* Willd. lyophilized extracts, obtained from two different samples, harvested from culture (TMC) and from spontaneous flora (TMW) on ambulatory activity and brain oxidative stress biomarkers on diabetic rats. The phenolic composition of lyophilized extracts was assessed by spectrophotometrical and LC-MS methods, while *in vitro* preliminary assays were carried out in order to assess the antioxidant capacity of tested samples. In addition, phospho-NFkB p65 subunit of transcription factor NFkB, histopathological changes; HDAC1 and MeCP2 expressions in the hippocampus and frontal lobe in rats with STZ experimental-induced hyperglycemia were investigated.

## Materials and Methods

### Reagents

2-thiobarbituric acid and EDTA-Na_2_ were obtained from Merck KGaA (Darmstadt, Germany), absolute ethanol and n-butanol were purchased from Chimopar (Bucharest, Romania). o-Phthaldehyde and Bradford reagent were obtained from Sigma–Aldrich Chemicals GmbH (Germany). Antibodies against HDAC1 (Cat# sc-56683, RRID : AB_783697), MeCP2 **(**Cat# sc-5755, RRID : AB_648930), glyceraldehyde 3-phosphate dehydrogenase (GAPDH) and secondary HRP-linked antibodies were purchased from Santa Cruz Biotechnology, Heidelberg, Germany. The test for quantification of phospho-NFkB p65 (Ser536) InstantOne™ ELISA was bought from Blue Gene, China. Glucose levels were measured by using a kit supplied by Diagnosticum Rt (Hungary). Chlorogenic acid, p-coumaric acid, caffeic acid, rosmarinic acid, rutin, apigenin, quercetin, isoquercitrin, quercitrin, hyperoside, kaempferol, myricetin, fisetin were purchased from Sigma Aldrich (St. Louis, USA). Ferulic acid, sinapic acid, gentisic acid, gallic acid, patuletin, luteolin from Roth (Karlsruhe, Germany) and cichoric acid, caftaric acid were obtained from Dalton (Toronto, Canada). HPLC grade methanol, ethanol and all reagents for spectrophotometric assays were purchased from Merck (Germany). All chemicals and reagents were of high-grade purity.

### Phytochemical Analysis of Samples

#### Plant Material

The flowering aerial parts of *T. marschallianus* Willd. were harvested in June 2016 from the spontaneous flora of North Eastern Moldavia Flora, Cricova surroundings (voucher No. 978, from which sample TMW was obtained). The cultured species was obtained from a culture initiated in the Experimental Fields of the Botanical Garden of the Moldavian Science Academy (voucher No. 979, from which sample TMC was obtained). The taxonomic identification of the species was performed by Nina Ciocârlan, PhD, from the Botanical Garden of the Moldavian Science Academy, where voucher specimens are deposited in the Herbarium (Niculae et al., 2019).

#### Preparation of the Lyophilized Extracts

The plant material was air dried at room temperature for 3 days and ground to a fine powder (300 μm). Extracts were prepared by maceration of the vegetal powder with 70% ethanol for 24 h, at room temperature. After filtration, the extracts were centrifuged at 4500 rpm for 15 min, and the supernatant was recovered and subjected to evaporation of the ethanol under a vacuum at 40°C, using a rotary evaporator. Afterwards, the extracts were transferred in glass vials and freeze-dried in a lab scale VirTis Advantage Plus freeze-drier (SP Scientific, Gardiner, USA) and lyophilized. The vials were placed on the freeze-dryer shelf and cooled to −50°C at a rate of 1°C/min. The temperature was kept constant for 10 h for the complete solidification of the extract. The primary drying was performed at −30°C for 48 h and vacuum of 0.2 mbar, followed by secondary drying at 20°C for 4 h at 0.2 mbar. After lyophilization, samples were stored at room temperature. For the HPLC determinations, TMC and TMW lyophilized extracts were dissolved in EtOH 70% at a concentration of 10 mg/mL. All assays were performed in triplicate (Olah et al., 2016).

#### Identification and Quantification of Polyphenolic Compounds

The phytochemical profile of lyophilized extracts was qualitatively and quantitatively analyzed by two LC-MS/MS methods assessing individual polyphenolic compounds.

The first LC-MS/MS method was used for the identification of 18 phenolic compounds such as caftaric acid, gentisic acid, caffeic acid, chlorogenic acid, p-coumaric acid, ferulic acid, sinapic acid, hyperoside, isoquercitrin, rutin, myricetin, fisetin, quercitrin, quercetin, patuletin, luteolin, kaempferol, and apigenin. The analytical method used a Zorbax SB-C18 chromatographic column for separation (100 mm x 3.0 mm i.d., 3.5 µm), while the mobile phase consisted of a mixture of methanol: 0.1% acetic acid (v/v). Compounds were eluted in a linear gradient, starting with 5% methanol and ending at 42% methanol at 35 min; for the next 3 min, isocratic elution was used, with 42% methanol; column was rebalanced in the next 7 min with 5% methanol. The flow rate was set at 1 mL/min, while the column temperature was set at 48°C. Injection volume was 5 µL. Detection of compounds was performed on UV and MS mode. The UV wavelength was set at 330 nm until 17 min (for the detection of polyphenolic acids), then at 370 nm until 38 min (to detect flavonoids and their aglycones). The MS system used an electrospray ion source in the negative mode (capillary +3000 V, nebulizer 60 psi (nitrogen), dry gas nitrogen at 12 L/min and dry gas temperature 360°C. The obtained chromatographic data were processed using ChemStation and DataAnalysis software from Agilent, USA. Identified polyphenols were quantified based on their peak areas and compared with a calibration curve of their corresponding references. Results were expressed as micrograms of polyphenolic compounds per gram of lyophilized extracts (Hanganu et al., 2016b; Ielciu et al., 2017; Ielciu et al., 2018).

Six polyphenols were detected and quantified by a second LC-MS method: epicatechin, catechin, syringic acid, gallic acid, protocatechuic acid, and vanillic acid. The same chromatographic column as in the first method was used. The mobile phase consisted of a mixture of methanol (A): 0.1% acetic acid (v/v) (B) with a binary gradient (0 min: 3% A; 0–3 min: 8% A; 3–8.5 min: 20% A; 8.5–10 min 20% A and finally a rebalance of the column with 3% A). The flow rate was set at 1 mL/min and the injection volume was 5 µL. Compounds were also identified by comparison of their retention times and the MS spectra with those of corresponding references, analyzed in the same chromatographic conditions (Rusu et al., 2018).

#### Quantification of Total Polyphenols, Flavonoids and Phenolic Acids Content

The total phenolic content (TPC) was spectrophotometrically determined by a method using the Folin-Ciocalteau reagent, according to the European Pharmacopoeia. 2.0 mL of each sample were mixed with 1.0 mL of Folin-Ciocalteu reagent, 10.0 mL of distilled water and the mixture was diluted to 25.0 mL with a 290 g/L solution of sodium carbonate. The absorbance was measured at 760 nm after 30 min. The calibration curve was made using as reference a calibration curve plotted using gallic acid. Gallic acid concentrations that were used were set at 0.02, 0.04, 0.06, 0.08, and 0.10 mg/mL and prepared in a mixture of methanol and water (50:50, v/v). TPC values were calculated using the calibration curve of gallic acid graph (R^2^ = 0.9953). Results were expressed as mg of gallic acid equivalents (GAE)/g of dry weight plant material (d.w) (Benedec et al., 2013a; Benedec et al., 2016b; Ielciu et al., 2019).

The quantitative determination of flavonoids (TFC) was performed by the spectrophotometric method using aluminum chloride. 5.0 mL of each sample were mixed with 5.0 mL of sodium acetate 100 g/L, 3.0 mL of aluminum chloride 25 g/L, and diluted to 25 mL by methanol in a calibrated flask. The absorbance was measured at 430 nm. Total flavonoids content values were determined using an equation obtained from calibration curve of the rutin graph (R^2^ = 0.9996). Results were expressed as mg of rutin equivalents (RE)/g d.w. (Benedec et al., 2013a; Benedec et al., 2016b; Ielciu et al., 2017).

The quantitative determination of phenolic acids (TPA) was analyzed spectrophotometrically, in a method according to the 10^th^ Edition of the Romanian Pharmacopoeia (*Cynarae folium* monograph), using Arnows reagent (10.0 g sodium nitrite and 10.0 sodium molybdate in 100 mL distilled water) and the results were expressed as mg of rosmarinic acid equivalents (RAE)/g of dry plant material, calculated using a rosmarinic acid calibration curve graph (R^2^ = 0.9985). All experiments were performed in triplicate. Results were expressed as mg of rosmarinic acid equivalents (RAE)/g d.w. (Benedec et al., 2013a; Benedec et al., 2016b; Ielciu et al., 2019).

### Antioxidant Activity Tests

#### DPPH Radical Scavenging Assay

The antioxidant potential of *T. marschallianus* Willd. samples were quantified using the stable DPPH radical method. The DPPH radical solution (0.1g/L) in methanol was prepared and 4.0 mL of this solution was added to 4.0 mL of extract solution (or standard) in methanol at different concentrations (10–50 μg/mL). After 30 min of incubation at 40°C in a thermostatic bath, the decrease in the absorbance (n = 3) was measured at 517 nm. The antiradical activity (three replicates per treatment) was expressed as IC_50_ (μg/mL), the concentration of vegetal material required to cause a 50% DPPH inhibition (Benedec et al., 2016a; Hanganu et al., 2016a; Ielciu et al., 2019).

#### FRAP Assay

This method evaluated the reduction of the iron, which is reduced from the ferric ion to the ferrous ion in a complex of iron with the radical 2,4,6-tripyridyl-s-triazine (TPTZ). The reduction of this ion is assessed by measuring the absorbance at 593 nm and the color change from green to yellow or blue. The FRAP reagent consists in a mixture of 2.5 mL 10 mM TPTZ solution in 40 mM hydrochloric acid to which 2.5 mL 20 mM ferric chloride solution and 25 mL acetate buffer at pH = 3.6 is added. At 0.4 mL of diluted sample, 6 mL the FRAP reagent was added and absorbance was measured. Blank consisted in the similar mixture without the sample. Results are expressed as mM Trolox equivalents/g dry weight vegetal material (d.w.), using a calibration curve (R^2^ = 0.989) constructed with 10-40 mg/L Trolox standard (Ielciu et al., 2018; Ielciu et al., 2019).

#### Electron Paramagnetic Resonance (EPR) Spectroscopy Method

EPR measurements were performed on a Bruker ELEXYS E-580 spectrometer with continuous wave at X band (~9.4 GHz, modulation amplitude, 1 G, microwave power, 9.6 mM, center field 3514 and sweep field 100 G, room temperature). A DPPH solution (4.5 mM) was mixed with 10 µL samples in liquid form and transferred in EPR quartz capillaries to record the EPR spectra at different time intervals. The rate of reaction between antioxidant samples and DPPH radical was expressed by integral intensity (I) and was obtained through double integration of experimental spectra using XEPR Bruker software (Mocan et al., 2014; Hanganu et al., 2016b).

### Animals and Experimental Design

**Figure 1:**
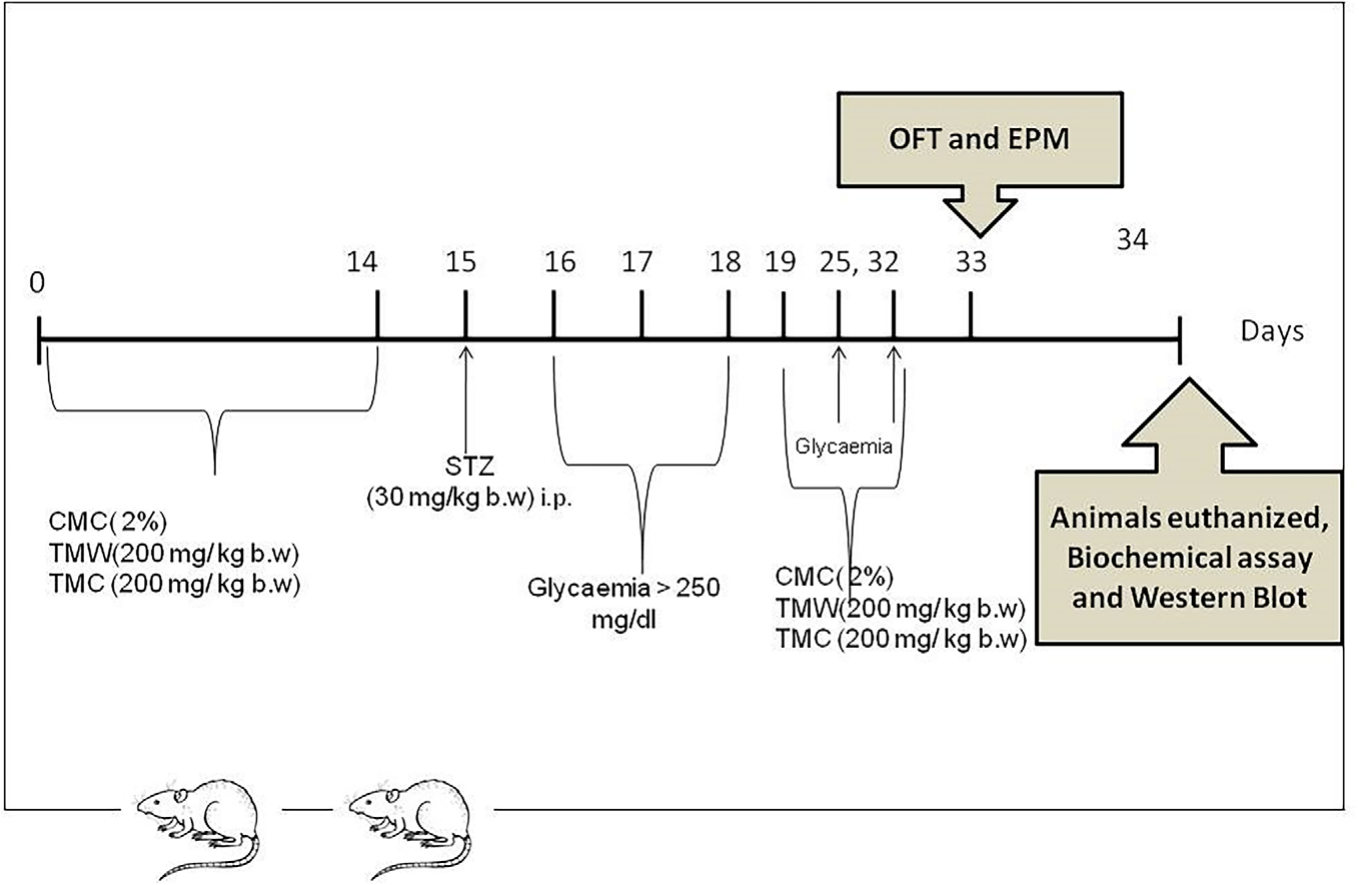
Experimental design: Four groups of 9 adult rats each were included in the study. One group received carboxymethyl cellulose (CMC) 2% for 14 days while TMW (group 3) and TMC (group 4), were orally administered, (200 mg/kg b.w.), dissolved in 0.5 mL CMC, for 14 days prior to DM induction. On the 15^th^ day, the animals from the three groups received one dose of STZ (30 mg/kg b.w.) administered intraperitoneally, in order to induce DM. Subsequently, CMC and the two extracts, dissolved in 0.5 mL CMC, were administrated for the next 14 days, between day 19 and 32. The results were compared with those of a control group treated with CMC without STZ (CMC). In this group CMC was administered in the same dose from day zero to day 32. On the 33^rd^ day, OFT and EPM were conducted. Twenty-four hours later, samples from the hippocampus and frontal lobe were collected for biochemical, ELISA test, western blotting analyzes and the frontal lobe for histopathological investigations.

Experimental procedures were approved by the Animal Ethics Board on animal welfare of the “Iuliu Haţieganu” University and by the Direction for Veterinary Surveillance and Food Safety according to the Directive 2010/63/EU on the protection of animals used for scientific purposes (Authorization No. 29/16.01.2017). Adult male Wistar rats (n = 36) were used under standard laboratory conditions, housed in a 12 h light–12 h dark cycle at room temperature (24 ± 2°C). The rats had free access to a standard normocaloric pellet diet (VRF1) and received water *ad libitum*. To evaluate the effects of TMC and TMW on brain oxidative stress biomarkers and ambulatory activity in rats with experimental-induced hyperglycemia, the animals were divided into 4 groups of 9 rats (**Figure 1**).

The group which received only carboxymethyl cellulose (CMC) 2% served as a control group (CMC). The animals from group 2 were given CMC for 14 days and streptozotocin (STZ) on the 15^th^ day (CMC + STZ). The other two groups of animals received: TMW respectively TMC in CMC for 14 days and STZ on the 15^th^ day, (CMC + STZ + TMW; CMC + STZ + TMC). TMW and TMC were administered orally 200 mg/kg body weight (b.w.), dissolved in 0.5 mL CMC and STZ in a dose of 30 mg/kg b.w. was administered intraperitoneally in the 15^th^ day (Opris et al., 2017). Fasting glycemia levels were assessed three times, on the 16^th^, 17^th^ and 18^th^ day of the experiment and DM was considered induced when blood glucose levels reached over 250 mg/dL. Subsequently, these two extracts and CMC were administrated for the next 14 days, between day 19 and 32. On the 33^rd^ day, OFT and EPM were conducted.

In literature, various doses of STZ can be used in order to experimentally induce diabetes mellitus, such as a single moderate dose, a single large dose or multiple low doses (Deeds et al., 2011; Damasceno et al., 2014; Qian et al., 2015; Nandini and Naik, 2019). Thus, based on the literature data (Qian et al., 2015) and on our previous experience (Opris et al., 2017), we chose 30 mg/kg b.w of STZ to induce hyperglycemia.

Twenty-four hours after the last behavioral test, under anesthesia with an intraperitoneal injection of ketamine/xylazine cocktail (90 mg/kg b.w. ketamine and 10 mg/kg b.w. xylazine), all animals were euthanized. The frontal lobe from 4 rats in each group was harvested for histopathological investigations. From the other 5 rats in the group, the hippocampus and frontal lobe were collected for oxidative stress assays, ELISA test and western blotting analyzes. Oxidative stress biomarkers in hippocampus and frontal lobe homogenates (malondialdehyde—MDA, glutathione reduced (GSH)/glutathione oxidized (GSSG) ratio and phosphorylated NFkB p65 in hippocampus and frontal lobe samples were also assessed. MECP2 and HDAC1 expressions in the rats’ brains were analyzed by Western Blot.

### Behavioral Testing

On day 33 of the study, two different tests (OFT and EPM) were used to assess the general locomotor activity and emotionality of the rodents. The animals` activity was quantified by a visual tracking system (Smart Basic Software version 3.0 Panlab Harvard Apparatus) using specific mazes for rats (Ugo Basil Animal Mazes for Video-Tracking).

#### OFT

The animals were freely allowed to explore an open field arena (100 × 100 × 40 cm) for 5 min. The total travelled distance and the total number of entered squares served as an index of general locomotor activity. Increases in central locomotion (number of entries and travelled distance in the center) or in time spent in the central part of the device (time spent in the center/total time) can be considered as anxiolytic-like behavior (Gamberini et al., 2015).

#### EPM

The plus-shaped maze consists of two open (10×50 cm) and two closed (10×50×40 cm) arms that are 60 cm elevated above the ground level. Although EPM is considered the gold standard for the evaluation of anxiety in the basic research, it also measures the motor activity. High open arms travelled distance, open arms number of entries and time ratio (open arms/total time) are considered relevant parameters of low anxiety-like behavior, whereas, total and closed arms travelled distance, total and closed arms entries are seen as an index of general locomotion in EPM. Between tasks, the mazes were cleaned with 70% ethanol to remove residual odor (Walf and Frye, 2007).

### Assessment of Glycemia

On the 16^th^ day of the study, retroorbital blood samples were collected to determine the glycemia levels, after one dose of STZ (30 mg/kg b.w.) administration. Rats with a glucose concentration exceeding 250 mg/dL, in the next 3 days after STZ injections, were considered diabetic. Later on, sugar blood levels were monitored on the 25^th^ and 32^nd^ days of the experiment.

### Biochemical Investigations of Oxidative Stress and Antioxidant Activity

For the oxidative stress evaluation, we measured malondialdehyde (MDA) as a marker of lipid peroxidation and GSH/GSSG ratio as an antioxidant parameter from the frontal lobe and hippocampus. The MDA levels in the hippocampus and frontal lobe were determined by spectrofluorimetry, using the 2-thiobarbituric acid method. The values were expressed as nmol/mL and nmoles/mg of protein (Conti et al., 1991). GSH and GSSG were measured fluorimetrically using o-phtalaldehyde in the two tissue homogenates. The values were expressed as GSH/GSSG ratios (Hu, 1994).

### Evaluation of HDAC1 and MeCP2 Expressions and Phosphorylated NFkB p65

HDAC1 and MeCP2 quantifications were performed by the Western Blot technique. Lysates (20 µg protein/lane) were separated by electrophoresis on 8% SDS PAGE gels under reducing conditions, then transferred to the polyvinylidenedifluoride membranes (BioRad), using V3 Western Workflow™ (BioRad). Blots were then blocked and incubated with antibodies against NFkB, phospho-NFkB HDAC1 (catalogue number SAB4503697) and MeCP2 (catalogue number M7443) (Santa Cruz Biotechnology, Heidelberg, Germany), diluted 1:500 and corresponding secondary HRP-linked antibodies (1:1500) (Santa Cruz Biotechnology). Proteins were visualized and detected using the Supersignal West Femto Chemiluminiscent substrate (Thermo Fisher Scientific, Rockford IL, USA) and a Gel Doc Imaging system equipped with an XRS camera and Quantity One analysis software (Biorad). GAPDH was used as a protein loading control. The phosphorylated NFkB p65 in the hippocampus and frontal lobe were measured by the ELISA technique (Blue Gene, China) following the manufacturer’s instructions. The results were expressed as OD/mg protein (Baldea et al., 2013).

### Histological Investigation of the Brain

Frontal lobe slices were harvested for histological investigation. Brain samples were fixed in 10% neutral buffered formalin, then embedded in paraffin in order to produce 5 µm thick sections which were stained with hematoxylin-eosin (HE) for light microscopy (Optika B-383LD2 microscope).

### Statistical Analysis

All statistical analyses were conducted using ANOVA GraphPad Prism software, version 6.0 (GraphPad, San Diego, California, USA) and SPSS v.11.5 for Windows. The results were expressed as the mean ± standard deviation (SD). One-way analysis of variance (ANOVA) was used, followed by Tukey’s *post hoc* test, to determine statistical significance among the four groups. A p value lower than 0.05 was considered statistically significant. Results are expressed as mean ± SD. For the extracts, differences between means were assessed by a Student–Newman–Keuls test (p≤ 0.05). Phytochemical assays were performed in triplicate. Each treatment contained three independent replicates and the experiments were performed twice.

## Results

### Phytochemical Characterization of TMW and TMC Lyophilized Extracts by HPLC-MS

The compound which was found in the highest amounts, both in the cultured sample and in the spontaneous one, is rosmarinic acid (RA), which appears therefore as a phenolic marker of the species. From the two tested samples, the one harvested from culture (TMC) appears to have a larger amount of RA. Another phenolic acid found in the composition of the species is ferulic acid, also having larger amounts in the cultured sample (TMC). Regarding the polyphenols, important amounts of aglycones and flavonoids were found, luteolin being the major one found in higher amounts in the cultured sample. Another polyphenolic compound identified in significant amounts in the lyophilized extracts was apigenin, having the opposite profile for the two tested samples, namely higher amounts in the spontaneous sample. Not least, kaempferol was found in larger amounts in TMW than in TMC (**Table 1**). Other compounds as catechin and protocatechuic acid, were also found in larger amounts in TMC than in TMW, while for syringic acid and vanilic acid, the amounts were reversed for the two samples (**Table 2**).

**Table 1:** Polyphenolic compounds analyzed by the LC-MS/MS method (I) in the lyophilized extracts.

Polyphenolic compounds	MonitoredIon (m/z)	Retention time (min)	Concentrations (µg/g lyophilized extract)	
			TMW	TMC
Gentisic acid	153	3.69 ± 0.04	*<*0.02	*<*0.02
Caffeic acid	179	5.60 ± 0.04	*<*0.02	*<*0.02
Chlorogenic acid	353	6.43 ± 0.05	*<*0.02	*<*0.02
*p*-Coumaric acid	163	9.48 ± 0.08	*<*0.02	*<*0.02
Ferulic acid	193	12.8 ± 0.10	64.86 ± 0.17*	142.01 ± 0.23*
Isoquercitrin	463	20.29 ± 0.10	*<*0.02	*<*0.02
Rutin	609	20.76 ± 0.15	*<*0.02	*<*0.02
Rosmarinic acid	360	20.8 ± 0.16	8542.14 ± 2.10*	11740.38 ± 3.5*
Quercitrin	447	23.64 ± 0.13	*<*0.02	*<*0.02
Quercetin	301	27.55 ± 0.15	27.59 ± 0.09	*<*0.02
Luteolin	285	29.10 ± 0.19	640.28 ± 1.09*	1105.57 ± 2.04*
Kaempferol	285	32.48 ± 0.17	66.23 ± 0.19*	59.38 ± 0.14*
Apigenin	279	33.10 ± 0.15	405.21 ± 1.01*	342.73 ± 0.91*

Values represent the mean ± standard deviations (n = 3), *p < 0.001.

**Table 2:** Polyphenolic compounds analyzed by the LC-MS method (II) in the lyophilized extracts.

Polyphenolic compounds	MonitoredIon (m/z)	Retention time (min)	Concentrations (µg/g lyophilized extract)	
			TMW	TMC
Catechin	289	6.0 ± 0.09	6.54 ± 0.02*	9.65 ± 0.04*
Syringic acid	197	8.4 ± 0.09	103.14 ± 0.19*	54.07 ± 0.09*
Protocatequic acid	153	2.8 ± 0.05	206.78 ± 0.41*	480.692 ± 0.83*
Vanilic acid	167	6.7 ± 0.07	270.65 ± 0.84*	67.27 ± 0.12*

Values represent the mean ± standard deviations (n = 3), *p < 0.001.

### Content of Total Polyphenols, Flavonoids and Phenolic Acid

Large amounts of total polyphenols, flavonoids and phenolic acids were determined for both samples. Small differences between the tested samples were noticed, except the content of flavonoids which appears to be almost double in the cultured sample (28.983 ± 0.32mg RE/g for TMC and 16.692 ± 0.51mg RE/g for TMW). The obtained amounts for this species are superior to the ones obtained from species belonging to other Lamiaceae species as *Ocimum basilicum* L. (Benedec et al., 2012) or *Rosmarinus officinalis* L. (Olah et al., 2016). *Origanum vulgare* L. and *Mentha* sp. showed superior results for TPC than *T. marschallianus* Willd., but inferior for TFC and TPA (Benedec et al., 2013b; Oniga et al., 2018).

### Antioxidant Activity

The antioxidant capacity of these extracts was determined by several methods, testing the behavior of these samples towards various radicals generated *in vitro*: DPPH bleaching assay, the ferric reducing antioxidant power assay (FRAP) and the electron paramagnetic resonance assay (EPR) (**Table 4**).

The DPPH scavenging ability of the TMC was 1.5 times higher than that of TMW (IC_50 =_ 81.2 ± 1.3 µg/mL and IC_50_ = 121.458 ± 1.21 µg/mL, respectively) (**Table 4**). This is in good connection with the TPC values listed in **Table 3**. Compared to the reference compounds, quercetin (IC_50_ = 5.40 ± 0.32 µg/mL) and BHT (IC_50_ = 15.6 ± 0.44 µg/mL), the extracts showed lower antioxidant capacity. Other studies also cite the antioxidant activity of *Thymus* species by this type of assay, but this biological activity is attributed to the essential oils (Jianu et al., 2015; Mancini et al., 2015; Petrović et al., 2017). For species belonging to the Lamiaceae family, results obtained in the same evaluation method range between 35.03 ± 1.57 and 135.89 ± 3.10 µg/mL (Benedec et al., 2015).

**Table 3:** The content of total polyphenols, flavonoids and phenolic acids for *T. marschallianus* Willd. Extracts.

Samples	TMC	TMW
**TPC (mg GAE/g d.w.)**	61.993 ± 0.31**	59.890 ± 0.42**
**TFC (mg RE/g d.w.)**	28.983 ± 0.32*	16.692 ± 0.51*
**TPA (mg RAE/g d.w.)**	26.512 ± 0.31**	25.484 ± 0.23**

Each value represents the mean ± standard deviations of three independent measurements, *p < 0.001, **p < 0.05.

GAE, Gallic acid equivalents; RE, Rutin equivalents, RAE, Rosmarinic acid equivalents.

**Table 4:** Antioxidant evaluation of *T. marschallianus* Willd.

Samples	TMC	TMW	Quercetin	BHT	DPPH
**DPPH** **(IC_50_ µg/mL)**	81.2 ± 1.32*	121.458 ± 1.21*	5.40 ± 0.32	15.6 ± 0.44	–
**FRAP** **(µM Trolox Eq/g d.w.)**	296.15 ± 4.73*	233.11 ± 3.53*	–	–	–
**EPR (I)**	218.04 ± 15.95*	341.50 ± 8.49*	–	–	797.01 ± 43.64

Each value represents the mean ± standard deviations of three independent measurements; I = integral intensity, *p < 0.001.

The FRAP assay shows values of 233.11 ± 3.5 µM Trolox Eq/g d.w for the TMW extract and of 296.15 ± 4.7 µM Trolox Eq/g d.w for the TMC extract, indicating a superior potential for the latter, in accordance with the TPC.

Regarding the EPR method, the values of the integral intensity of these samples were compared with the DPPH radical standard, which were mixed. The rate of reaction between the antioxidant compounds of the extracts and DPPH radical was monitored using normalized double integrated residual EPR signal, correlated with the number of paramagnetic species. It is therefore observed that the integral intensity of DPPH in mixture with samples decreases, especially compared with the DPPH solution without samples. Decrease of the integral intensity for the tested samples can be observed, representing by the oxido-reduction rate of the DPPH radical. Comparing the obtained rates for both samples, it is clear that TMC has a higher antioxidant capacity than TMW. Values of the integral intensity of both samples are represented in **Table 4** compared with DPPH (Mocan et al., 2014).

All of the obtained results in these assays are in correlation with the content of polyphenols, flavonoids and phenolic acids and also with the amounts of the compounds quantified by the HPLC method. These results provide important directions on possible mechanisms of actions of these samples, taking into consideration the different systems that were used, which test the behavior of these compounds towards various radicals. These assays represented the premises that allowed to further test the effects of oxidative stress in the model of STZ-induced hyperglycemia in rats.

### Behavioral Studies

**Figure 2:**
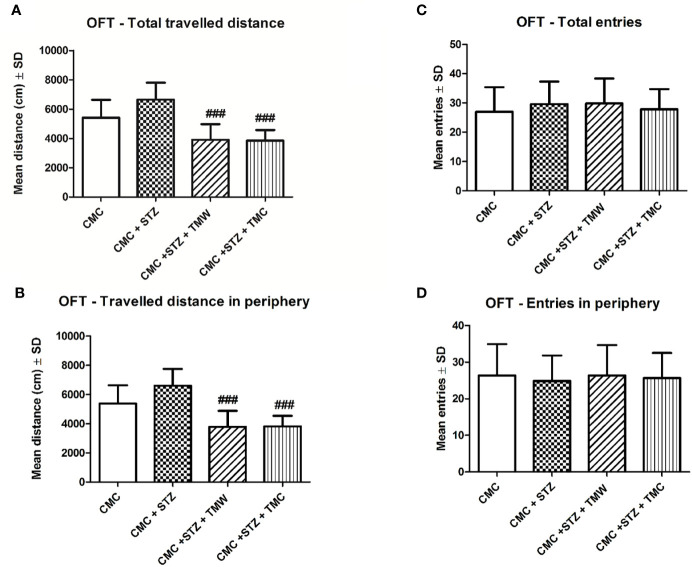
The effects of two extracts on the total **(A)** and peripheral **(B)** travelled distance and total **(C)** and peripheral **(D)** number of entries in the open field test (OFT). TMW and TMC (p<0.001) significantly diminished the total travelled distance **(A)** and the travelled distance in periphery **(B)** as compared to the CMC+STZ group. Twenty-eight days of extracts treatment in comparison to STZ administration, slightly improved the locomotor activity (number of entries in periphery), but without a statistical significance (p > 0.05) **(D)**. Each group consisted of 9 rats. Results are expressed as mean ± SD; p < 0.05 as compared to CMC+STZ. The ^###^ means p<0.001 between CMC+STZ versus treated groups (CMC+STZ+TMW and CMC+STZ+TMC).

The effect of extracts on locomotion of rats, tested in OFT is illustrated in **Figure 2**. The OFT is used to assay general locomotor activity levels and anxiety in rodents in scientific studies (Walf and Frye, 2007; Anchan et al., 2014; Kushwah et al., 2016). Our results showed that TMW and TMC (p<0.001) significantly diminished the total travelled distance and travelled distance in periphery.

**Figure 3:**
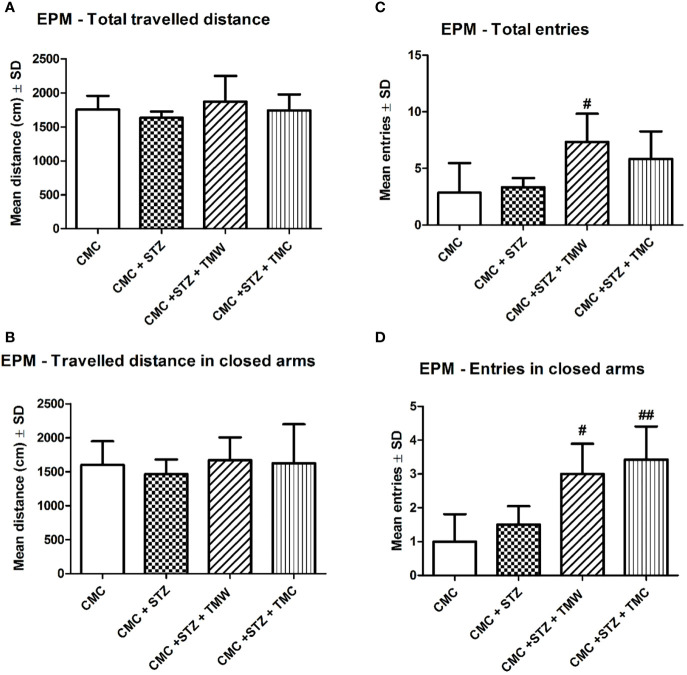
The effects of extracts on the total **(A)** and peripheral **(B)** travelled distance and the total **(C)** and peripheral **(D)** number of entries in the elevated plus maze (EPM). In the EPM, the natural compound administration improved general locomotion, but without any statistical significance (p > 0.05) **(A, B)**. TMW **(C, D)** and TMC **(D)** treated rats significantly made more entries in the EPM test as compared to the CMC+STZ group (p < 0.05). Each group consisted of 9 rats. Results are expressed as mean ± SD; ^#^p < 0.05 as compared to CMC+STZ. The ^##^ means p<0.01 between CMC+STZ versus treated groups (CMC+STZ+TMW and CMC+STZ+TMC).

The effect of these two extracts on the locomotion of rats, tested in EPM is illustrated in **Figure 3**. Even though, EPM was developed to measure anxiety-related behavior, this test is used to assay general locomotor activity levels (number of entries into the open arms and total arms entries), as well (Walf and Frye, 2007).

**Figure 4:**
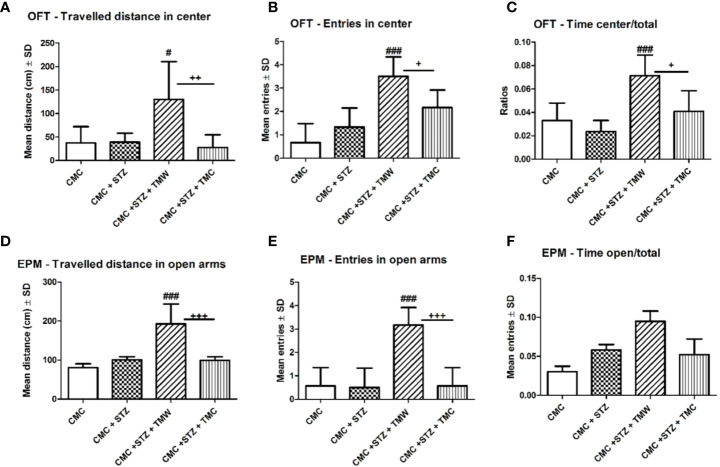
The effects of extracts on emotionality in the open field test (OFT) **(A–C)** and in the elevated plus maze (EPM) **(D–F)**. The TMW treated rats travelled significantly greater distance (p < 0.05) **(A)**, made more entries (p < 0.001) **(B)**, and spent more time (p < 0.001) **(C)**, in the central part of the OFT arena as compared to CMC+STZ. TMW administration increased the central travelled distance (p < 0.01) **(A)**, the number of entries (p < 0.05) **(B)**, and the central time spent (p<0.05) **(C)**, in the open filed arena as compared to the CMC+STZ+TMC group. In EPM, TMW administration enhanced the travelled distance and the entries made in the open arms, both as compared to the CMC+STZ and CMC+STZ+ TMC group **(D, E)** (p < 0.001). TMW group tended to increase the time spent in the open arms of the EPM, but without statistical significance (p > 0.05). Each group consisted of 9 rats. Results are expressed as mean ± SD; ^#^p < 0.05 as compared to CMC+STZ; ^+^p < 0.05 as compared to CMC +STZ+TMW. The ^###^ means p<0.001 between CMC+STZ versus treated groups (CMC+STZ+TMW and CMC+STZ+TMC). ^++^p<0.01 and ^+++^p<0.001 between CMC+STZ+TMW and CMC+STZ+TMC.

In the EPM, the extracts administration improved general locomotion, but without any statistical significance (p>0.05). TMW and TMC treated rats significantly made more entries in the EPM test as compared to the STZ group (p< 0.05). The influence of the administration of extracts on the emotionality, tested in OFT and EPM, was exemplified in **Figure 4**. Regarding the emotionality in OFT, the TMW treated rats travelled significantly greater distance (p<0.05), made more entries (p<0.001), and spent more time (p <0.001) (C), in the central part of the OFT arena as compared to STZ. TMW administration increased the central travelled distance (p<0.01), the number of entries (p<0.05), and the central time spent (p< 0.05), in the open filed arena as compared to the TMC group.

EPM has been validated to assess the anxiety-like behavior in rats, based on the natural aversion of rodents for open spaces of the elevated maze (Wang et al., 2014). Thus, higher travelled distance, more entries and more time spent in the open arms of the EPM test apparatus, during a 5 min test session, is indicative of low anxiety-like behavior (Walf and Frye, 2007).

In EPM, the TMW group exhibited a significantly higher travelled distance and made more entries in the open arms, both as compared to the CMC+STZ and CMC+STZ+TMC group (p<0.001). Moreover, the TMW group tended to increase the time spent in the open arms of the EPM, but without statistical significance (p>0.05).

### Assessment of Glycemia

**Figure 5:**
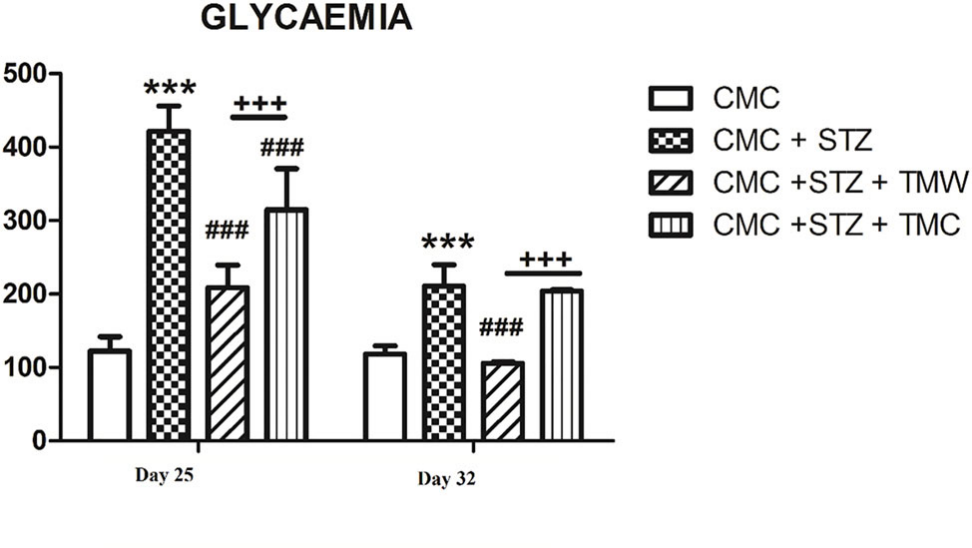
The effects of the extracts on glycemia levels in the blood of rats. Both on 25^th^ and 32^nd^ days of the experiment, glycemia displayed higher levels in the CMC+STZ group as compared to the CMC group (p<0.001). On the 25^th^ day, both TMW and TMC decreased glycemia values (p<0.001), while on the 32^nd^ day, TMW was the only one to exert beneficial effects (p<0.001). Significantly higher levels of glycemia were recorded in the CMC+STZ+TMC group as compared to the CMC+STZ+TMW group (p<0.001). Each group consisted of 9 rats. Results are expressed as mean ± SD; ***p<0.001 as compared to CMC; ^###^p<0.001 as compared to CMC+STZ; ^+++^p<0.001 as compared to CMC+STZ+TMW.

The effects of administration of extracts on glycemia levels in the blood of the rats are exemplified in **Figure 5**. On the 25^th^ and 32^nd^ days of the experiment, glycemia levels were measured in the blood of the rats. On both days, glycemia displayed higher levels in the CMC+STZ group as compared to the CMC group (p<0.001). On the 25^th^ day, both TMW and TMC decreased glycemia values (p<0.001), while on the 32^nd^ day, TMW was the only one to exert beneficial effects (p<0.001). Significantly higher levels of glycemia were recorded in the CMC+STZ+TMC group as compared to the CMC+STZ+TMW group (p<0.001) (**Figure 5**), which is possibly due to higher content of total polyphenols in the composition.

### Oxidative Stress and Antioxidant Activity Assessment in the Hippocampus and Frontal Lobe

**Figure 6:**
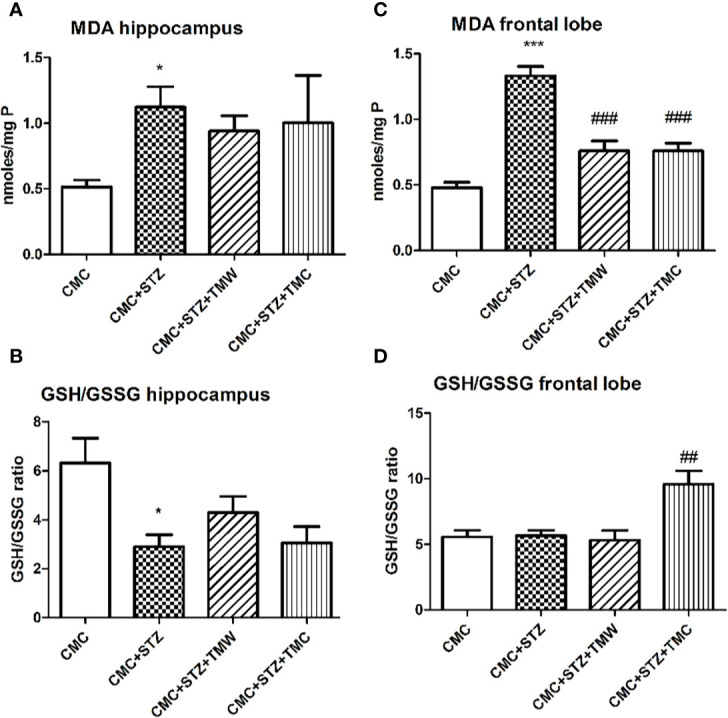
The effects of two extracts on malondialdehyde (MDA) levels **(A, C)** and antioxidant defense (GSH/GSSG ratio), **(B, D)** in the hippocampus and frontal lobe of adult rats. MDA displayed higher levels in the hippocampus and frontal lobe of the CMC+STZ group (p < 0.05, **A**; p < 0.001, **C**). Both TMW (p < 0.001) and TMC (p < 0.001) diminished the MDA levels in the frontal lobe **(C);** GSH/GSSG ratio was significantly lower in hippocampus (p<0.05) **(B)** of the CMC+STZ treated group. TMC administration significantly increased the GSH/GSSG ratio in the frontal lobe of the treated rats compared to the untreated animals (p < 0.01) **(D)**. Each group consisted of 5 rats. Results are expressed as mean ± SD; *p<0.01 and ***p<0.001 as compared to CMC; ^##^p<0.01 and ^###^p<0.001 as compared to CMC+STZ.

The effects of extracts administration on oxidative stress markers in different areas of the rats’ brains are found in **Figure 6**.

### Evaluation of Phosphorylated NFkB p65, HDAC1, and MeCP2 Expressions

**Figure 7:**
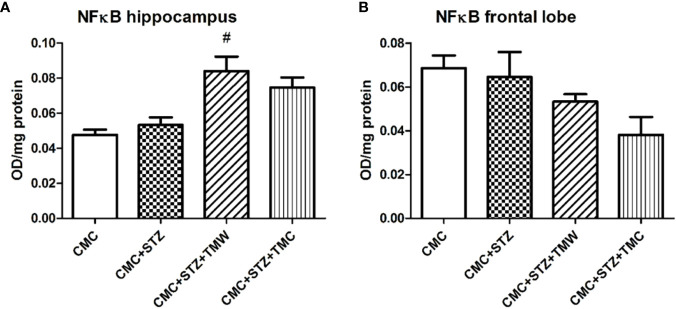
The effects of the extracts on NFkB levels in the brains of the rats **(A, B)**. NFkB recorded elevated levels in the hippocampus of the CMC+STZ+TMW treated group as compared to CMC+STZ (p < 0.05) **(A)**. In the frontal lobe, the NFkB levels were down-regulated both by TMW and TMC, but the differences were not statistically significant (p > 0.05) **(B)**. Each group consisted of 5 rats. Results are expressed as mean ± SD; ^#^p < 0.05 as compared to CMC+STZ.

The effects of extracts on the phosphorylated NFkB p65 subunit, in the brain of the rats are illustrated in **Figure 7**. The phosphorylation of p65 NFkB at Ser 536 allows the nuclear localization of the transcriptionally active complex and transactivation of several downstream genes mediated by NFkB. Therefore, the quantification of active form of p65NFkB subunit is an indirect measure of NFkB activation and function. Phospho-NFkB p65 increased in the hippocampus of the CMC+STZ+TMW treated group as compared to CMC+STZ (p<0.05). In the frontal lobe, the phospho-NFkB p65 were down-regulated both by TMW and TMC, but the differences were not statistically significant (p>0.05). The levels of the phospho-NFkB p65 decreased 1.28 times in TMW groups as compared to CMC and 1.21 times as compared to STZ. The levels of the phospho-NFkB p65 decreased 1.79 times in TMC group as compared to CMC and 1.69 times as compared to STZ.

**Figure 8:**
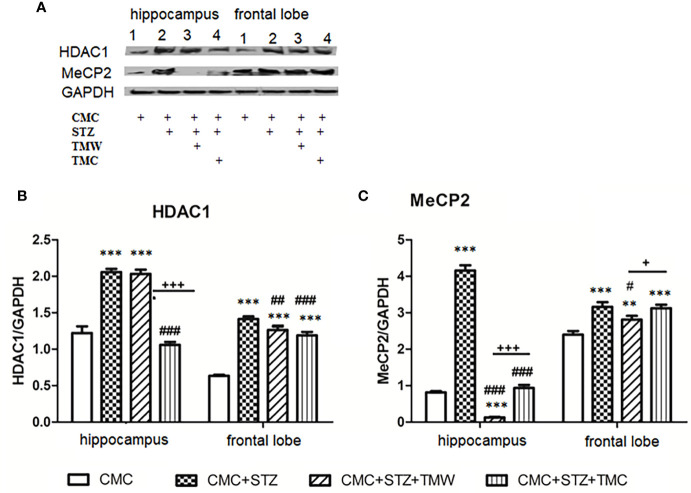
The effects of the administration of extracts on the expression of HDAC1 and MeCP2 in the brain **(A–C)**. Expression of HDAC1 and MeCP2 were analyzed by western blot (WB). The image analysis of western blot bands was completed by densitometry; the results were normalized to GAPDH. WB images, 1 - 4 hippocampus, (1 = CMC; 2 = CMC + STZ, **3** = CMC + STZ + TMW; 4 = CMC + STZ + TMW) 1 – 4 frontal lobe, (**1** = CMC; 2 = CMC + STZ, 3 = CMC + STZ + TMW; 4 = CMC + STZ + TMW); (n = 3). Each group consisted of 3 samples. Results are expressed as mean ± SD; **p<0.01 and ***p<0.001 as compared to CMC; ^#^p<0.05, ^##^p<0.01 and ^###^p<0.001 as compared to CMC+STZ; ^+^p<0.05 and ^+++^p<0.001 as compared to CMC+STZ+TMW.

The influence of the administration of extracts on the expression of HDAC1 and MeCP2 expressions in the brain is shown in **Figure 8**.

STZ in CMC significantly stimulated the HDAC1 and MeCP2 levels both in the hippocampus and the frontal lobe as compared to the control group (p<0.001). TMW administration significantly lowered the HDAC1 value in the frontal lobe (p<0.01) (**Figure 8B**) and MeCP2 both in the hippocampus (p<0.001) and the frontal lobe (p<0.05) as compared to STZ in CMC (**Figure 8C**). TMC decreased the expressions of HDAC1 in the hippocampus and the frontal lobe (p<0.001) (**Figure 8B**) and of the MeCP2 in the hippocampus (p<0.001).

**Figure 9:**
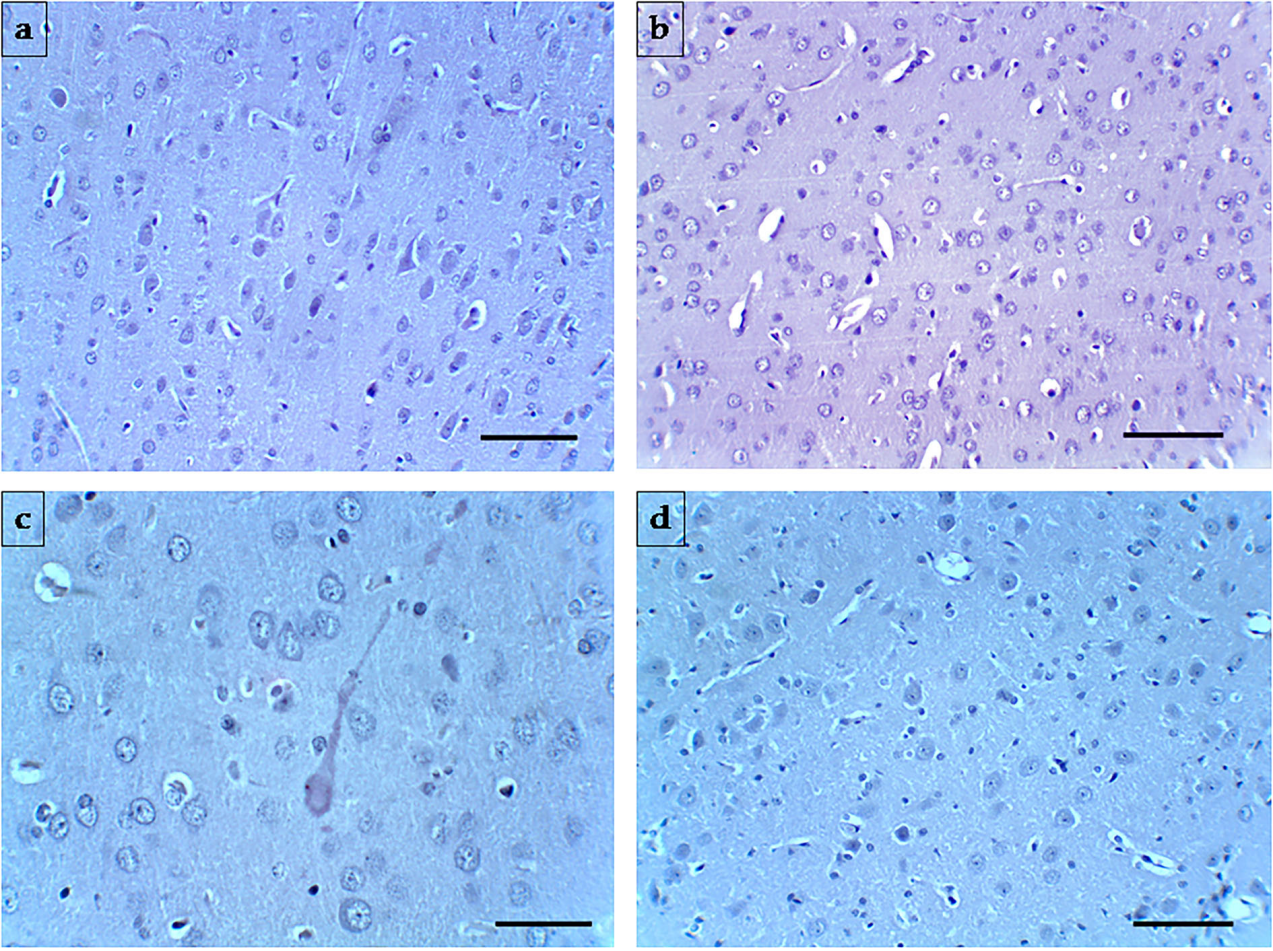
Representative photomicrographs of the frontal cortex of the four experimental groups. **(A)** (CMC), **(B)** (CMC+STZ), **(C)** (CMC+STZ+TMW), **(D)** (CMC+STZ+TMC) prove the histological features of the frontal cortex Magnification: ×200. H&E staining. Scale bar = 20 µm.

The effects of extracts administration on the micromorphology of the frontal cortex are illustrated in **Figure 9**. The tissue sections evaluated by morphometry displayed rare perineuronal edema in the frontal cortex of CMC group. Frequent perineuronal edema, shrinked neurons along with axonal demyelination was observed in the CMC+STZ treated group. TMW and TMC administration induced rare perineuronal edema, a few shrinked neurons along with rare axonal demyelination in the frontal lobe.

## Discussion

Herbal medicines are used for the treatment and prevention of various diseases starting from ancient times. Among the disorders that were treated with natural remedies DM is also included (Petrovska, 2012; Choudhury et al., 2018). Numerous natural products have shown their efficiency in reducing blood glucose levels, regulating insulin secretion and insulin sensitivity in the cells and reducing triglycerides and cholesterol levels. Therefore, many patients consider complementary and alternative medicinal (CAM) therapies over conventional ones, due to their lower costs and less side effects, medicinal plants still being used with great interest in the modern era (Choudhury et al., 2018).

In the present study, we have chosen a chemical model to induce experimental diabetes in animals, which is based on STZ administration. STZ (2-deoxy-2-(3-(methyl-3- nitrosoureido)-D-glucopyranose), synthesized by *Streptomycetes achromogenes*, has been widely used for its diabetogenic properties in rodents, either in a single or multiple dose injection. STZ causes DNA alkylation while entering the pancreatic β cells *via* the GLUT2 glucose transporter, which promotes the activation of poly ADP-ribosylation that further leads to cellular NAD^+^ and ATP reduction and subsequent inhibition of insulin synthesis and secretion. Moreover, it is well known that STZ – induced DNA damage may be also related to nitric oxide (NO) delivery, a molecule released during STZ cellular metabolism (Takasu et al., 1991; Bedoya et al., 1996; Szkudelski, 2001; Deeds et al., 2011). Results of our research provided evidence that STZ (30 mg/kg b.w.) administration increased the oxidative stress parameters in the brain of the hyperglycemic rats. Thus, we observed enhanced MDA levels in the hippocampus and the frontal lobe, and reduced GSH/GSSG ratio in the hippocampus. These observations were consistent with studies that reported high lipid peroxidation, enhanced protein carbonyl content, as well as altered enzymatic activity (decreased glutathione peroxidase, superoxide dismutase, catalase), and reduced glutathione levels in hypothalamus, hippocampus and frontal cortex lysate of STZ - treated rats (Parihar et al., 2016).

There is scientific evidence that diabetes mellitus may consistently be associated with reduced cognitive performance, especially in elderly people. The exact link between diabetes mellitus and cognitive deficits is not fully elucidated, but some authors have revealed that insulin and the insulin-like growth factor (IGF) regulate neuronal and glial cell activities (e.g. growth, survival, metabolism, gene expression, protein synthesis, cytoskeletal assembly, synapse formation, neurotransmitter function, and plasticity), which are highly needed to promote cognitive function (Kodl and Seaquist, 2008; De la Monte, 2014). Conversely, brain insulin/IGF resistance induces oxidative stress, neuroinflammation, impaired cell survival, mitochondrial dysfunction, dysregulated lipid metabolism, and endoplasmic reticulum (ER) stress, thus propagating a mild neurodegenerative process, similar to Alzheimer’s disease (AD) (Tong and De La Monte, 2009; De la Monte, 2014).

Some authors revealed that polyphenol administration may prevent neurodegeneration, inhibit inflammations and reduce age – related cognitive decline by scavenging free radicals, activating various signaling pathways, modulating gene expression, down-regulating NFkB and nuclear factor erythroid 2-related factor 2 (Nrf2) or inhibiting the release of cytokines (IL-1β, TNF-α) (Spencer et al., 2009; González-Gallego et al., 2010; Vauzour, 2012; Vauzour, 2017). In the CNS, NFkB transcription factors may play an important regulatory role in physiological processes, such as neurogenesis, neuritogenesis and synaptic plasticity which is related to learning and memory. Moreover, Nrf2 and NFkB are considered to regulate cellular responses to oxidative stress and inflammation, both being activated by similar stimuli. Based on scientific evidence, the absence of Nrf2 can exacerbate NFkB activity leading to increased cytokine production, whereas NFkB can modulate Nrf2 transcription and activity, having both positive and negative effects on the target gene expression (Wardyn et al., 2015). Thus, the functional cross-talk between the Nrf2 and NFkB pathways may lead to the development of improved therapeutic strategies that may regulate the NRF2-NFkB interplay response under both physiological and pathological conditions (Sivandzade et al., 2019).

The comparative effects of two lyophilized extracts of *T. marshallianus*, harvested from the wild flora (TMW) and from the culture (TMC) on STZ – induced hyperglycemia were studied in this experimental model due to the background of the *Thymus* species present in traditional medicine, but moreover, as large amounts of rosmarinic acid (RA) were found in the composition of the tested samples (**Table 1**). In the context of the well-known protective activities of this compound (Nunes et al., 2017; Nadeem et al., 2019), the promising potential of this species proved to be significant and therefore the present study aimed to highlight its potential medicinal properties.

The results of the present study evidenced that administration of both TMW and TMC diminished lipid peroxidation, whereas TMC improved the antioxidant activity in the frontal lobe of the STZ – treated rats. These observations were consistent with the literature. Umeno et al. (2016) mentioned that polyphenols may exert an antioxidant effect by Nrf2 pathway activation and by antioxidative proteins expression (e.g. HMOX-1). Among the polyphenols, RA is mentioned by scientific literature to decrease serum MDA levels and improve CAT, SOD, GSH, GPx, tumor necrosis factor-alpha (TNF)- α and interleukin-6 (IL-6). The antioxidant mechanism of the extracts can also be related to this compound, known as the most potent scavenger of ROS, RNS and peroxynitrite identified among polyphenols (Nadeem et al., 2019).

Based on the quantitative determinations of polyphenols by HPLC, tested samples revealed a high content of RA, which is proven to be the major compound found in the composition of the species. Together with the RA, significant amounts of flavonoids were identified. Taken into consideration the background of these compounds (Bamosa et al., 2010; Maithili Karpaga Selvi et al., 2015), they may be involved in the glucose-metabolism pathways, such as, glucose absorption, regulation of glucose production in the liver or insulin tissue sensitiveness. Considering that hyperglycemia induces oxidative stress and increased levels of inflammatory mediators and apoptotic proteins associated with diabetes mellitus, these extracts might be considered for both antioxidant and hypoglycemic effects.

The results showed that rats injected with STZ showed elevated plasma glucose levels, an observation also reported by other authors (Yassa and Tohamy, 2014), whereas blood glucose levels were significantly reduced in diabetic rodent groups treated with natural compounds. Hence, our data were in agreement with previous studies that have shown the hypoglycemic efficacy of polyphenolic compounds (Chen et al., 2015).

Regarding the behavioral effects of TMW in our study, there was a decrease in the general activity, revealed by significantly lower scores in the peripheral and total travelled distance, assessed in OFT. However, based on the EPM test, administration of TMW improved the general locomotion (increased zone transition number and entries in closed arms) and TMC increased the entries in the closed arms. Additionally, TMW demonstrated an anxiolytic-like effect in OFT as it enhanced the travelled distance, the entries made and the time spent in the central part of the arena. Further, the TMW treated rats made more entries and travelled a higher distance in the open arms of the EPM as compared to the STZ treated group.

In addition, we evaluated the HDAC 1 and MeCP2 expressions in the hippocampus and frontal lobe by western blot. Histone deacetylases, (HDACs), a family of four classes enzymes, divided into zinc (*class I:* HDACs 1, 2, 3 and 8; *class II:* HDACs 4, 5, 6, 7, 9 and 10; *class IV*: HDAC 11) and nicotinamide adenine dinucleotide - dependent groups (*class III* HDACs: sirtuins), play a major role in the normal cellular brain activities by regulating, gene expression, survival or proliferation of the cells (Reddy et al., 2018; Gatla et al., 2019). Class I HDACs, expressed in various mammalian cells and tissues, have been intensively studied as histone modifiers and transcriptional repressors (Jaworska et al., 2015). Moreover, the importance of both HDAC1 and 2 in the development of CNS, as *hdac 1* mutation induced neuron and glia failure cell formation in the hindbrain, loss of segmental organization of postmitotic neurons and glia cells and deficit in the branching of motor neurons in zebrafish (Pillai et al., 2004; MacDonald and Roskams, 2008). It also seems that the deletion of both HDAC 1 and 2 may impair the normal development of the mouse brain (e.g. cortical, hippocampal and cerebellar structures) during the embryonic period, leading to neuronal death (Haberland et al., 2009; Montgomery et al., 2009).

HDAC 1 showed significant expression in glial and neural progenitor cells during brain development, and in glial cells in adult brain, whereas HDAC 2 was expressed in progenitors and neurons (Broide et al., 2007; MacDonald and Roskams, 2008). HDACs, by histone deacetylation, remove acetyl groups from N-terminal tails of histone proteins, being involved in the epigenetic modifications that stabilize the local chromatin architecture and thus decrease gene expression and protein function (Jaworska et al., 2015; Khurana and Dlugos, 2017; Dresselhaus et al., 2018).

Moreover, histone acetylation is involved in the memory formation. Thus, the high levels of acetylated hippocampal histones in mice and the efficiency of HDAC inhibitors (e.g. trichostatin A, TSA, VPA, vorinostat), that may facilitate the learning process in wild-type mice, as well as in neurodegeneration (Guan et al., 2009; Ricobaraza et al., 2012; Peixoto and Abel, 2013; Lu et al., 2015). At the same time, Groh et al. (2013) mentioned that polyphenols, such as epigallocatechin-3-gallate and genistein potently diminished the activity of HDAC in intact colon carcinoma cells and demonstrated that the modulation of HDAC activity is associated with the suppression of HDAC1. In our study, one dose of STZ increased the HDAC1 and MeCP2 expressions both in the hippocampus and the frontal lobe. TMW administration significantly inhibited the expression of HDAC1 in the frontal lobe and MeCP2, both in the hippocampus and the frontal lobe. TMC downregulated the expressions of HDAC1 in the hippocampus and frontal lobe and of MeCP2 in the hippocampus, this being in agreement with the above-mentioned data.

MeCP2, a member of the methyl-CG-binding domain (MBD) family of proteins, may repress transcription in a histone deacetylase (HDAC)-dependent process (e.g. binding of MeCP2 to Sin3A-HDAC complexes), and in a HDAC-independent manner (e.g. inhibiting of the assembly of transcription preinitiation complexes and MeCP2 functioning as a global methyl-CG-specific, histone deacetylase independent repressor) (Wan, 2001; Fuks et al., 2003; Clouaire and Stancheva, 2008; Theisen et al., 2013). However, scientific data sustained that MeCP2 can induce either repression or activation of gene transcription, processes that highly depend on the cellular and molecular context (Hansen et al., 2010; Theisen et al., 2013).

Despite the significant use of HDAC inhibitory agents (HDACi), such as valproic acid (VPA) in nervous system disorders (e.g. epilepsy), the HDACs expression in the central nervous system remains still unclear (MacDonald and Roskams, 2008; Khurana and Dlugos, 2017). Decreased HDAC2 protein expression in the nucleus accumbens was also associated with both depression in humans and chronic stress-like behavior in mice (Massart et al., 2012). Additionally, it has been reported that MeCP2 overexpression in mice neurons may be associated with anxiety, impaired memory and learning, abnormal motor coordination and abnormal hippocampal synaptic plasticity due to a HDAC repressed–transcription–mechanism. Moreover, previous evidence sustains that HDAC co-repressor proteins may negatively regulate NFκB transcriptional activity (Ashburner and Westerheide, 2001).

As NFkB modulates ROS levels and the expression of proinflammatory factors (interleukin (IL)-1, intercellular adhesion molecule 1, tumor necrosis factor α (TNF α), therefore, it can be considered to initiate the inflammatory cascade (Wang et al., 2019). Previous studies also revealed that HDACi inhibits NFkB transcription, thus repressing inflammatory response (Lu et al., 2015). Additionally, it was suggested that high blood glucose and oxidative stress levels may lead to neuroinflammation *via* NFκB signaling (Richa et al., 2017). Thus, we also evaluated the active form of p65 subunit, phospho-NFkB p65 expressions in the hippocampus and frontal lobe. In our study, both TMW and TMC tended to decrease the phospho-NFkB p65 in the frontal lobe and consequently the NFkB transcriptional activity. Conversely, in the hippocampus, with regard to administration of extracts, our results were contradictory with the above-mentioned data. These observations are consistent with the literature. Several authors reported that polyphenols administration reduced the expression of NF-κB in in different brain areas (e.g. hippocampus, striatum and frontal cortex) and cell types (astrocytes and microglia) (Vauzour, 2012; Sarubbo et al., 2017; Jha et al., 2019).

It is known that the transcription factor NFκB may exhibit cellular context-dependent function in the nervous system (Kaltschmidt and Kaltschmidt, 2009). NF-κB is involved in multiple physiological and pathological processes, (e.g. neurotransmission and neuroprotection, inflammation, immunity, apoptosis, cell proliferation, differentiation and survival) and can be activated by various factors: inflammatory cytokines, antigen receptor engagement, UV- or γ-irradiation, ischemia and hyperosmotic shock or oxidative stress (Meffert et al., 2003; Hayden et al., 2006; Oeckinghaus and Ghosh, 2009). In unstimulated cells, IκB kinase (IKK) complex can induce nuclear translocation of inactive NFκB and transcription of target genes. The constitutive active form of NFkB can be found in mature B cells, macrophages, neurons, vascular smooth muscle cells and tumoral cells (Oeckinghaus and Ghosh, 2009).

Although significant progress has been made in the understanding of the NFkB activation mechanism, the functional significance of this process in the nervous system still requires further research. Several authors sustain that NFkB can either regulate cell death or survival in the nervous system, depending on the type of cell (e.g. glia vs neurons), different stimuli or molecular context. To elucidate the influence of natural products on NFkB activation and its role in the nervous system, further studies should be performed (Kaltschmidt and Kaltschmidt, 2009; Mincheva-Tasheva and Soler, 2013). Our data showed that diabetic rats had no significant degree of histological brain damage, thus sustaining biochemical and functional - related damages rather than morphological ones.

## Conclusions

Our study demonstrated that TMW and TMC administration reduced blood glucose levels. TMW improved the central locomotion of the rats, both in OFT and EPM. In the frontal lobe, both extract diminished lipid peroxidation and HDAC1 expression but enhanced the antioxidant capacity. TMW administration increased the phospho-NFkB p65 and diminished MECP2 expression in the hippocampus. Our findings indicate that administration of *Thymus marschallianus* Willd. extracts (either TMW or TMC extracts) might represent a good option in diabetes-related complications, by exerting various beneficial effects *via* several/various mechanisms. Both extracts exerted a beneficial effect by increasing the antioxidant defense and improving the central locomotion.

## Data Availability Statement

The raw data supporting the conclusions of this article will be made available by the authors, without undue reservation, to any qualified researcher.

## Ethics Statement

The animal study was reviewed and approved by Animal Ethics Board on animal welfare of the “Iuliu Hatieganu” University and by the Direction for Veterinary Surveillance and Food Safety.

## Author Contributions

All authors have contributed directly and intellectually to the study. AS-B, II, GF, IO, DB, IC, SC and DH contributed to the design of the study, writing the original draft and revising it critically. AS-B, II, LV, AOM, A-MG, VT, BM, AM, IB, DO have contributed to the experimental part of the study. All authors have read and approved the final form of the manuscript for publication.

## Funding

This work was supported by a grant of the Romanian Ministry of Education and Research, CNCS - UEFISCDI, project number PN-III-P1-1.1-PD-2019-0967, within PNCDI III.

## Conflict of Interest

The authors declare that the research was conducted in the absence of any commercial or financial relationships that could be construed as a potential conflict of interest.
